# Adjuvant anti-PD-1 therapy improves melanoma-specific survival in stage IIIC-IV melanoma patients with high tumor mutation burden and *BRAF V600* mutation

**DOI:** 10.3389/fonc.2025.1618596

**Published:** 2025-08-12

**Authors:** Julia Forschner, Julia Huynh, Christopher Schroeder, Sorin Armeanu-Ebinger, Olga Seibel-Kelemen, Axel Gschwind, Irina Bonzheim, Thomas K. Eigentler, Teresa Amaral, Stephan Ossowski, Lukas Flatz, Claus Garbe, Andrea Forschner, Markus Reitmajer

**Affiliations:** ^1^ Department of Dermatology, University Hospital Tübingen, Tübingen, Germany; ^2^ Department of Dermatology, Charité - Universitätsmedizin Berlin, Corporate Member of Freie Universität Berlin and Humboldt-Universität zu Berlin, Berlin, Germany; ^3^ Institute of Medical Genetics and Applied Genomics, University Hospital Tübingen, Tübingen, Germany; ^4^ Institute of Pathology and Neuropathology, University Hospital Tübingen, Tübingen, Germany

**Keywords:** adjuvant immune checkpoint inhibitors, relapse-free survival, melanoma-specific survival, tumor mutational burden, *BRAF V600E/K* mutation

## Abstract

Immune checkpoint inhibitors (ICI) have significantly improved melanoma-specific survival (MSS), particularly in patients with tumors with a high tumor mutational burden (TMB) or *BRAF* mutation. In the adjuvant setting, ICIs significantly improve relapse-free survival (RFS), but data on MSS are still lacking. Tissue samples from 83 patients with stage IIIC/D/IV melanoma who started adjuvant ICI between March 2018 and September 2019 were examined using a 700 gene panel. TMB and *BRAF V600E/K* mutation status were analyzed to determine their potential influence on RFS and MSS. TMB levels ≥ 20 Var/Mb were classified as TMB high, corresponding to the top 20% TMB levels in the cohort. RFS and MSS were significantly improved in patients whose tumors had high TMB levels and *BRAF V600E/K* mutation (p<0.001 and p=0.002, respectively). Patients with *BRAF*-mutated tumors and high TMB seem to benefit particularly from adjuvant ICI.

## Introduction

With the introduction of immune checkpoint inhibitors (ICIs), progression-free survival and melanoma-specific survival (MSS) have improved significantly in advanced melanoma (1, 2). Combined ICIs are recognized as one of the most effective treatments for metastatic or locally advanced melanoma, with a 10-year MSS rate exceeding 50% in patients treated with first-line ipilimumab and nivolumab ICI (1–3).

However, more than half of the patients receiving combined ICI develop severe treatment-related adverse events and only 40-50% of patients respond to ICI (2, 4). Numerous studies have been conducted to determine potential predictive factors of response to ICI. Tumor-specific factors, such as high tumor mutational burden (TMB) and the presence of a *BRAF* mutation were found to be associated with improved outcome with ICI in the non-adjuvant setting (2, 4–8).

In the adjuvant setting, ICI have also been proven to significantly improve relapse-free survival (RFS) and distant metastasis free survival (DMFS) (9–12). Furthermore, for the CheckMate 238 study, numerically, but not significantly, overall survival was increased with adjuvant anti-PD-1 treatment compared to adjuvant CTLA-4 treatment (10, 11). Recently, the presence of *BRAF* mutations and high TMB levels were found to be associated with improved RFS in the adjuvant setting with anti-PD-1 treatment (1, 10, 13).

To date, no data demonstrate significant improvements in MSS with adjuvant ICI in advanced melanoma. In a previous study, we reported a significantly improved RFS in a cohort of patients with high-risk melanoma at stage IIIC-IV who received adjuvant ICI. RFS was longer in patients whose tumors had a *BRAF* mutation and a high TMB (13). With an extended follow-up (FU) of up to five years, we report MSS data from this cohort and examine potential correlations between MSS, *BRAF* mutation status, and TMB level.

## Material and methods

### Eligibility criteria and ethical approval

We included all patients with stage IIIC, IIID and IV melanoma who started adjuvant anti-PD-1 therapy at our dermato-oncology department between 01/03/2018 and 30/09/2019, and had tumor tissue available for next-generation sequencing (NGS). As NGS of tumor tissue is more difficult in tumors with low tumor content, such as in stage IIIA and IIIB patients, we only included stage IIIC-IV patients.

The recommendation for adjuvant treatment was based on the German melanoma guidelines and was made by the institutional interdisciplinary tumor board (14).

Demographic and clinical information, including sex, age, melanoma subtype, date of primary diagnosis, stage at the start of adjuvant anti-PD-1 therapy, and details of recurrences (location, type and timing) were extracted from the electronic patient file.

This study complies with the Declaration of Helsinki and was approved by the local Ethics Committee of the University of Tübingen (project number 606/2020BO). All patients included in the study gave written informed consent for the documentation of their clinical data for research purposes and publication.

### Next-generation sequencing

NGS was performed on tumor- and normal tissue at the Institute for Medical Genetics and Applied Genomics at the University Hospital Tuebingen. DNA was isolated from tumor FFPE tissue and blood samples using the Maxwell^®^ RSC DNA FFPE kit and the Maxwell^®^ RSC instrument (Promega, Madison, WI, USA) according to the manufacturer’s standard protocols. After isolation, 200 ng of genomic DNA was sequenced using a Covaris ultrasonicator (Covaris, Woburn, MA, USA) and target regions were captured and enriched using the SureSelect XT Low Input Target Enrichment System (Agilent Technologies, Santa Clara, CA, USA). The sequencing panel included 708 cancer-related genes, 7 promoter regions and selected fusion sites.

The sequencing data were analyzed using the in-house bioinformatics pipeline meg-SAP (https://github.com/imgag/megSAP, https://github.com/imgag/ngs-bits). Sequencing reads were aligned to the human reference genome GRCh37 using BWA MEM (15). Small somatic variants (SNVs) and insertion/deletion (indels) were identified using Strelka2 (16) and annotated using variant effect predictor (VEP) (17). ClinCNV was used to detect somatic copy number variants (18). TMB was calculated according to the methodology described previously by Forschner et al. (2020) (19). A ‘high’ TMB was defined as the top 20% of the cohort according to Samstein et al. (2019) (20), which corresponded to TMB levels of ≥ 20 variations per megabase (Var/Mb) in this study.

### Statistical analysis

Statistical analyses were performed using IBM^®^ SPSS^®^ Statistics 28 (IBM, Armonk, USA). TMB and *BRAF* mutation status, sex, stage at the start of adjuvant anti-PD1- treatment and the occurrence of immune-related adverse events were analyzed in view of their potential influence on RFS and MSS using univariate Cox regression analysis. Factors that were significant or near significant in the univariate Cox regression analysis were also tested in a multivariate Cox regression analysis. RFS was defined as the time between administration of the first cycle of anti-PD-1 antibody and recurrence, melanoma-specific death, or censoring on the last date of patient contact. MSS was defined as the time between the first application of anti-PD-1 antibody and melanoma-specific death or censoring on the last date of patient contact.

In addition, combined variables were created to analyze the potential influence of TMB and *BRAF* mutation status (TMB-high + *BRAF* mutation, TMB-high + *BRAF* wildtype, TMB-low + *BRAF* mutation, and TMB-low + *BRAF* wildtype). According to Samstein et al. (20) we classified the top 20% of the cohort as ‘high’ TMB, which in this study corresponded to TMB levels of at least 20 variants per megabase (Var/Mb). RFS and MSS were analyzed according to these combined variables using Kaplan-Meier estimator. Differences between groups were tested for significance using the log-rank-test, with a significance threshold of 0.05 (two-sided). Thus, p-values < 0.05 were considered as significant.

## Results

### Patient characteristics

A total of 165 patients started adjuvant anti-PD-1 therapy between March 1, 2018, and September 30, 2019, at the dermato-oncology outpatient department of the University Hospital Tübingen. Of these, 85 patients had melanoma at stage IIIC-IV. Two patients were excluded because NGS of the tumor tissue could not be successfully performed. Consequently, 83 patients were included in this study: 35 female patients (42%) and 42 male patients (58%). The median age at the start of anti-PD-1 therapy was 65 years, interquartile range (IQR) was 55.5–77 years (**Table 1**). Further information can be found in **Table 1** and **Supplementary Table 1**.

**Table 1:** Patient characteristics.

Patients characteristics				n	%
Sex					
	Female			35	42
	Male			48	58
**Age at start of anti-PD-1 therapy in years (median, IQR)**				65 (55.5-77)	
**Follow-up in months (median, IQR)**				59 (32-66)	
**Anti-PD-1 therapy**				83	100
	Nivolumab 480 mg every 4 weeks			78	94.0
	Pembrolizumab 400 mg every 6 weeks			4	4.8
	Pembrolizumab 200 mg every 3 weeks			1	1.2
**Time to first relapse in months (median, IQR)**				6 (3-12.5)	
Melanoma subtype					
	Cutaneous			62	75
	Occult			12	14.5
	Acral			5	6
	Mucosal			4	5
Breslow thickness					
	≤ 2.0			12	14.4
	2.1-4.0			23	27.7
	>4			36	43.4
	Unknown			12	14.5
Ulceration					
	Yes			40	48.2
	No			34	41.0
	Unknown			9	10.8
Stage at start of adjuvant anti-PD1 therapy					
	Stage IIIC			68	82
	Stage IIID			5	6
	Stage IV			10	12
Status (MSS)					
	Alive			54	65
	Dead			29	35
Status (RFS)					
	No relapse			34	41
	Relapse			49	59
		Distant relapse	N= 23		
		Locoregional relapse	N= 26		
Immune-related adverse event					
	No			62	75
	Yes			21	25

The median FU time was 59 months (IQR 32–66 months). Cutaneous melanoma was the most common subtype (n=62, 75%), followed by occult melanoma (n=12, 15%), acral melanoma (n=5, 6%), and mucosal melanoma (n=4, 5%). At the beginning of adjuvant anti-PD-1 therapy, most patients (n=68; 81,9%) were stage IIIC. Five patients (6%) had stage IIID melanoma and 10 patients (12%) had stage IV melanoma with no evidence of disease (**Table 1**).

After five years of FU, most patients were still alive (n=54, 65%), and 29 patients died of melanoma (35%). The median time to first relapse was 6 months (IQR 3-12.5 months). A total of 34 patients (41%) remained relapse-free, and 49 patients (59%) relapsed, of which 23 patients had a distant relapse and 26 patients had a locoregional relapse. Immune-related adverse events occurred in 21 patients (25%) (**Table 1**).

### TMB and BRAF mutation status had a significant effect on RFS and MSS

**Figure 1:**
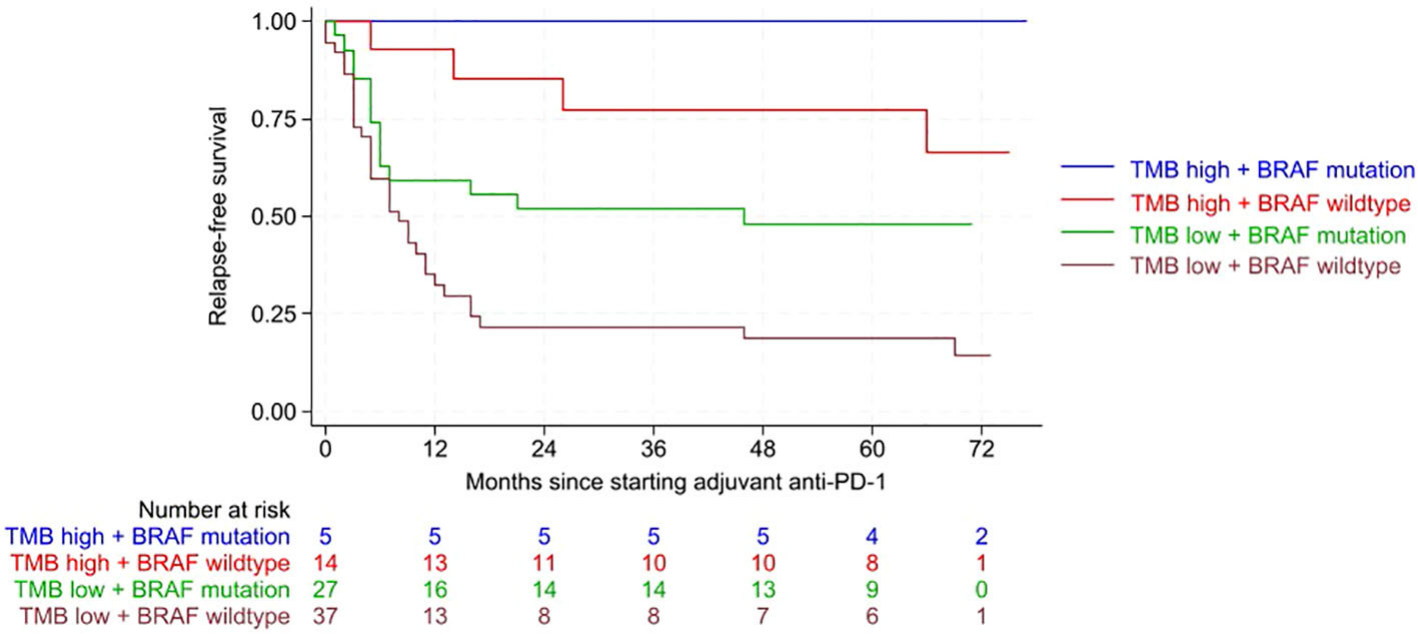
Kaplan-Meier curves showing relapse-free survival (RFS) according to the combined variables of tumor mutational burden (TMB) and *BRAF* mutation. RFS was significantly improved for tumors with high TMB and presence of a *BRAF* mutation (p<0.001). Following abbreviations are used in the graph: Tumor mutational burden (TMB), anti-Programmed Death-1 (anti-PD-1).

RFS was significantly improved in tumors with high TMB and presence of *BRAF* mutation (p<0.001) (**Figure 1**). Multivariate Cox regression analysis revealed a significantly higher risk for relapse in *BRAF* wild-type tumors than in *BRAF*-mutated tumors (HR 2.279; CI 1.218-4.427; p= 0.010). Tumors with low TMB were at a significantly higher risk for relapse compared to high TMB tumors (HR 6.240; CI 2.222-17.519; p<0.001). Both factors, TMB and *BRAF* mutation status, were independent significant factors influencing RFS in the multivariate Cox regression analysis.

**Figure 2:**
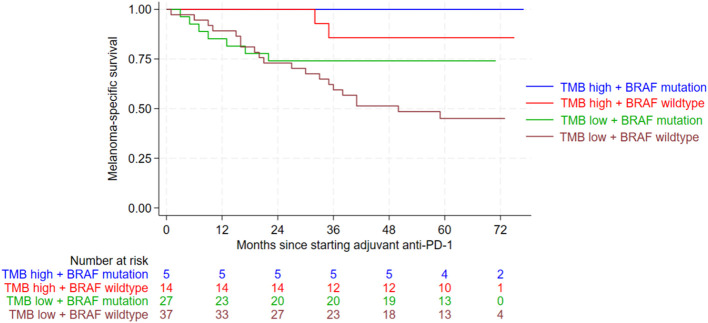
Kaplan-Meier curves showing melanoma-specific survival (MSS) according to the combined variables of tumor mutational burden (TMB) and *BRAF* mutation. MSS was significantly improved for patients with BRAF mutation and a high TMB level (p=0.002). Following abbreviations are used in the graph: Tumor mutational burden (TMB), anti-Programmed Death-1 (anti-PD-1).

In terms of MSS, patients whose tumors had a *BRAF* mutation and a high TMB had significantly better outcomes (p=0.002) (**Figure 2**). In multivariate Cox regression analysis absence of *BRAF* mutation doubled the risk of death from melanoma (HR 2.341; CI 0.997-5.498; p=0.051). Even more important, however, is that multivariate Cox regression analysis revealed a significantly increased risk of death from melanoma in tumors with low TMB levels compared to those with high TMB (HR 5.696; CI 1.349-24.055; p=0.018). This means that TMB is a strong and independent influencing factor on MSS. The risk to die from melanoma is about 6-fold increased for patients with TMB-low (HR 5.7). In case of *BRAF* wildtype, the risk to die from melanoma was doubled (HR 2.3). Other patient characteristics, such as sex, tumor stage at the start of adjuvant PD-1 treatment, and the occurrence of immune-related adverse events, did not have a significant impact on RFS or MSS in the univariate Cox regression analysis (**Supplementary Tables 2**, **3**).

## Discussion

Adjuvant ICI provides significant improvements in RFS and DMFS in patients with stage III melanoma (9, 11). However, despite a FU times of seven years, no significant improvement in MSS has been achieved with adjuvant ICI so far (9).

In this study, we identified significant tumor-specific influence factors associated with improved RFS and MSS in patients with stage IIIC-IV melanoma. The strongest influence factor was a high TMB level, that was independent in multivariate testing for MSS and RFS. The combination of TMB high and presence of *BRAF* mutation was associated with the best MSS and RFS.

In the non-adjuvant setting, a positive correlation between *BRAF* mutation status and survival has already been observed with combined ipilimumab and nivolumab treatment (2). Other studies have revealed a positive association between high TMB values and improved outcomes in advanced melanoma treated with ICI (8, 21). In the adjuvant setting, of the CheckMate 238 study, high TMB and *BRAF*-mutated tumors were associated with improved RFS (10). In a previous publication of this cohort, we reported a significantly improved RFS for patients with high TMB and presence of *BRAF* mutation (13). However, data on improved MSS for *BRAF*-mutated tumors and high TMB were lacking. To our knowledge, this is the first study to report significantly improved MSS after adjuvant ICI in patients with *BRAF*-mutated tumors and high TMB.

The risk of melanoma relapse or death was approximately twice as high for *BRAF* wildtype tumors and an even stronger influence was found for TMB. The risk of melanoma recurrence or death was almost six times higher for tumors with low TMB

In our study, 59% of the patients relapsed, 31% locoregional and 28% distant. Comparing these results with those of the KEYNOTE-054 study, we had a higher overall relapse rate. In the KEYNOTE-054 study, there were 244 relapses, corresponding to 47% of the cohort receiving adjuvant pembrolizumab. The percentage of patients with distant metastasis was approximately one third (28%) in our cohort, which is similar to the pembrolizumab arm of the KEYNOTE-054 study (28%) and the nivolumab arm of the CheckMate 238 study (27%). The rate of locoregional relapses in our study was twice (31%) as high than in the KEYNOTE-054 study (15%) and the nivolumab arm of the CheckMate 238 study (17%) (9, 10).

The higher recurrence rate in our cohort can be explained with the exclusion of stage IIIA and IIIB melanoma patients and the consequently higher percentage of stage IIIC-IV melanoma patients included in our study. The KEYNOTE-054 study enrolled patients with stage IIIA or IIIB melanoma, while the CheckMate 238 study included patients with stage IIIB melanoma. As a result, both studies focused also on patients with lower risk of relapse. The percentage of distant metastases was almost identical to that observed in these two studies. However, the percentage of locoregional relapses was twice as high compared to the KEYNOTE-054 and CheckMate 238 study. It has to be considered, that the FU time in the KEYNOTE-054 study was longer with seven years, compared to our study, which had a FU of five years. Therefore, another FU at a later time point may have further increased the difference.

The positive correlation between high TMB and *BRAF* mutation on MSS in patients treated with ICI has already been described in the non-adjuvant setting (4, 8). Therefore, our results confirm the data of the non-adjuvant setting in the adjuvant setting treated with ICI. Additional factors have been identified as predictive markers for patients undergoing adjuvant treatment, such as T and NK cell subsets as negative predictive indicators (22) or the presence of circulating tumor DNA (ctDNA) post-surgery (23). In this cohort of advanced melanoma patients, we considered two factors, that were easy to obtain: TMB and *BRAF* mutation status based on the NGS results. However, a multifactorial approach, incorporating more predictive factors could further enhance the significance of the findings. Furthermore, it cannot be ruled out whether tumors with a high TMB value have a better prognosis independent of ICI, i.e. TMB may not only be a predictive marker for response to ICI (20), but also be generally regarded as a favorable prognostic factor (24). Although the *BRAF* mutation status is a well-known marker that predicts the response to BRAF- and MEK-targeted therapies, its potential as an additional prognostic marker should be considered, particularly in the adjuvant setting. Indeed, the presence of a *BRAF* mutation has been discussed in several publications as a potential negative prognostic factor in melanoma (25, 26).

It should also be considered, that we only report on adjuvant ICI and not on adjuvant targeted therapy. Most recently, two retrospective, real-world studies have been published, indicating that in the case of a *BRAF* mutation, adjuvant BRAF MEK inhibitor therapy with dabrafenib and trametinib may be superior to adjuvant ICI (27, 28). Our study does not answer the question of whether one adjuvant treatment regime is superior to the other. However, our findings suggest that TMB high tumors may derive particularly benefit from adjuvant ICI. Prospective trials are needed to evaluate whether TMB-high, BRAF-mutant patients benefit more from adjuvant BRAF/MEK inhibition or from adjuvant ICI.

Another aspect as to be considered due to most recently published studies:

in the case of macrometastasis, a neoadjuvant-adjuvant approach with pembrolizumab according to the SWOG regime (29) or a neoadjuvant treatment with 2 cycles of combined ipilimumab and nivolumab according to the NADINA regime has been proven to be superior to adjuvant-only procedures (30). However, neoadjuvant ICI are not approved in this indication and can usually not be carried out without prior clarification of costs with the health insurance companies.

It is the strength of our study that all patients included were treated within one single center, thereby minimizing the potential bias that could arise from variations in follow-up procedures or medical documentation. Additionally, the use of a 700-gene sequencing panel enabled a robust and accurate calculation of TMB values. However, it is important to consider the limited sample size of or cohort with even smaller numbers in each subgroup. Nevertheless we found out, that patients with stage IIIC-IV melanoma and low TMB values were six times more likely to relapse or die from melanoma than those with high TMB values. Similarly, patients with *BRAF* wildtype tumors had twice the risk of relapse or death from melanoma compared to patients with *BRAF*-mutated tumors. In patients with TMB-low and *BRAF* wild-type tumors, adjuvant radiotherapy could be recommended with a higher priority and close follow-up including ultrasound and more frequent radiologic staging intervals may be reasonable to be able to detect relapse as early as possible. For the future, larger and prospective studies are needed to verify our results and also to find out whether TMB-high, *BRAF*-mutant patients benefit more from adjuvant BRAF/MEK inhibition or from adjuvant ICI.

## Author’s Note

This study had been selected for poster presentation at the 11th World Congress of Melanoma in Athens in April 2025. Therefore parts of this work had been presented there.

## Data Availability

The raw data supporting the conclusions of this article will be made available by the corresponding author, without undue reservation.
