# Supplementary material for: Adjuvant anti-PD-1 therapy improves melanoma-specific survival in stage IIIC-IV melanoma patients with high tumor mutation burden and BRAF V600 mutation
Source: Front Oncol. 2025 Aug 12;15:1618596. doi: 10.3389/fonc.2025.1618596 (PMC12378509; doi:10.3389/fonc.2025.1618596)
Supplement: Supplementary file 1 [file DataSheet1.docx]

**Supplementary Tables**

| **Second line treatments** | **N** | **%** |
| --- | --- | --- |
| Surgery only | 5/49 | 10.2 |
| Anti-PD1-therapy (adjuvant, post-surgery) | 16/49 | 32.7 |
| BRAF/MEK inhibitors (adjuvant, post-surgery) | 3/49 | 6.1 |
| ICI (non-adjuvant)* | 17/49 | 34.7 |
| BRAF/MEK inhibitors (non-adjuvant) | 3/49 | 6.1 |
| CTX (non-adjuvant) | 2/49 | 4.1 |
| Study therapy (non-adjuvant) | 3/49 | 6.1 |

**Supplementary Table 1 Second line treatments**

Supplementary Table 1 shows the second-line treatments received by all 49 patients who experienced a relapse.The following abbreviations are used: Anti-programmed cell death protein 1 therapy (anti-PD-1 therapy), BRAF and MEK inhibitors (BRAF/MEK inhibitors), immune checkpoint inhibitors (ICI), and chemotherapy (CTX). Three patients receiving ICI in the non-adjuvant setting additionally received intralesional therapy: two with Talimogene laherparepvec (T-VEC) and one with Interleukin-2 (IL-2) (*).

| **Characteristic** | | **n** | ***P*** |
| --- | --- | --- | --- |
| **Sex** | |  |  |
|  | Female | 35 | 0.859 |
|  | Male | 48 |  |
| **BRAF-Mutation Status** | |  |  |
|  | BRAF wildtyp | 51 | **0.056** |
|  | BRAF V600 mutation | 32 |  |
| **TMB high-low** | |  |  |
|  | TMB low | 64 | **0.001** |
|  | TMB high | 19 |  |
| **Stage at start of adjuvant PD-1 therapy** | |  |  |
|  | Stage IIIC | 68 | 0.912 |
|  | Stage IIID/IV | 15 |  |
| **Immune-related adverse event** | |  |  |
|  | No | 62 | 0.621 |
|  | Yes | 21 |  |

**Supplementary Table 2 Univariate Cox regression analysis of RFS according to patient-specific factors.** The p-values in bold indicate which variables were used in the multivariate cox regression analysis

| **Characteristic** | | **n** | ***p*** |
| --- | --- | --- | --- |
| **Sex** | |  |  |
|  | Female | 35 | 0.466 |
|  | Male | 48 |  |
| **BRAF-Mutation Status** | |  |  |
|  | BRAF wildtyp | 51 | **0.104** |
|  | BRAF V600 mutation | 32 |  |
| **TMB high-low** | |  |  |
|  | TMB low | 64 | **0.027** |
|  | TMB high | 19 |  |
| **Stage at start of adjuvant PD-1 therapy** | |  |  |
|  | Stage IIIC | 68 | 0.514 |
|  | Stage IIID/IV | 15 |  |
| **Immune-related adverse event** | |  |  |
|  | No | 62 | 0.718 |
|  | Yes | 21 |  |

**Supplementary Table 3 Univariate Cox regression analysis of MSS according to patient-specific factors.** The p-values in bold indicate which variables were used in the multivariate cox regression analysis
